# Strategies for Introducing *Wolbachia* to Reduce Transmission of Mosquito-Borne Diseases

**DOI:** 10.1371/journal.pntd.0001024

**Published:** 2011-04-26

**Authors:** Penelope A. Hancock, Steven P. Sinkins, H. Charles J. Godfray

**Affiliations:** Department of Zoology, University of Oxford, Oxford, United Kingdom; The University of Queensland, Australia

## Abstract

Certain strains of the endosymbiont *Wolbachia* have the potential to lower the vectorial capacity of mosquito populations and assist in controlling a number of mosquito-borne diseases. An important consideration when introducing *Wolbachia-*carrying mosquitoes into natural populations is the minimisation of any transient increase in disease risk or biting nuisance. This may be achieved by predominantly releasing male mosquitoes. To explore this, we use a sex-structured model of *Wolbachia-*mosquito interactions. We first show that *Wolbachia* spread can be initiated with very few infected females provided the infection frequency in males exceeds a threshold. We then consider realistic introduction scenarios involving the release of batches of infected mosquitoes, incorporating seasonal fluctuations in population size. For a range of assumptions about mosquito population dynamics we find that male-biased releases allow the infection to spread after the introduction of low numbers of females, many fewer than with equal sex-ratio releases. We extend the model to estimate the transmission rate of a mosquito-borne pathogen over the course of *Wolbachia* establishment. For a range of release strategies we demonstrate that male-biased release of *Wolbachia*-infected mosquitoes can cause substantial transmission reductions without transiently increasing disease risk. The results show the importance of including mosquito population dynamics in studying *Wolbachia* spread and that male-biased releases can be an effective and safe way of rapidly establishing the symbiont in mosquito populations.

## Author Summary

*Wolbachia* are symbiotic bacteria that are found in many insect species. Recent laboratory studies show that certain strains of *Wolbachia* can reduce the capacity of mosquito species to transmit diseases such as dengue fever and malaria, either by directly inhibiting the pathogen or by shortening lifespan. However, little is known about how easily these bacteria will spread in natural mosquito populations or the impact of deliberate *Wolbachia* introduction on disease transmission. We use a simple model of *Wolbachia*-mosquito interactions to explore the design of field releases of infected mosquitoes to initiate symbiont spread. A particular concern is how *Wolbachia* can be introduced while releasing only small numbers of female mosquitoes which may bite humans and transmit disease. The models include explicit mosquito population dynamics including seasonal fluctuations in population size and different forms of population regulation. We find that rapid *Wolbachia* establishment is possible by releasing predominantly male mosquitoes, though the number of insects introduced may need to be large. This strategy requires the introduction of considerably fewer females compared to equal sex-ratio releases and is unlikely to increase disease transmission throughout the intervention. We demonstrate that once *Wolbachia* has become established, substantial reductions in disease transmission are possible.

## Introduction

Mosquito-borne parasites and viruses cause some of the world's most important diseases, disproportionately affecting poor communities and representing a major public health challenge. Biological control techniques aimed at suppressing mosquito populations or reducing their capacity to transmit disease may be a useful addition to traditional vector control strategies, especially if resistance to chemical insecticides in mosquito populations continues to rise [1]. Recently there has been increased interest in the use of certain strains of *Wolbachia* bacteria to reduce transmission by mosquito vectors of human diseases [2]–[7]. *Wolbachia* are maternally-inherited endosymbiotic bacteria that are common in many insect species including mosquitoes. *Wolbachia* spread in mosquito populations by manipulating the host's reproduction using a mechanism known as cytoplasmic incompatibility (CI) [8]. CI occurs when *Wolbachia* in infected males modify the sperm of their host such that arrest of embryonic development occurs unless the egg also carries the bacterium. Uninfected females are therefore at a disadvantage, and the *Wolbachia* spreads by a process of positive frequency-dependent selection. Models of *Wolbachia* dynamics show that spread will occur if the proportion of infected hosts exceeds a threshold that is higher for *Wolbachia* that cause stronger reductions in host fitness [9].

Recent studies indicate that infecting mosquito populations with certain strains of *Wolbachia* may lower their rates of disease transmission for two reasons. First, the bacteria may reduce mean adult lifespan [6], [10]. Because most vector-borne pathogens have a relatively long extrinsic incubation period in the mosquito a reduction in average longevity disproportionately affects infectious individuals, with beneficial consequences for disease transmission [11], [12]. However, a reduction in longevity also lowers the fitness of *Wolbachia* carriers and hence increases the threshold infection frequency required for spread to occur [13]. An ideal strain would increase mortality only late in life as this would (i) particularly affect pathogen-carrying individuals; (ii) have a lesser effect on host fitness and thus require fewer individuals to be introduced to pass the threshold infection frequency; and (iii) lead to less selection for modulation of the harmful effects of these *Wolbachia*. Second, *Wolbachia* can inhibit the development, replication or dissemination of important mosquito-borne pathogens, including filarial nematode parasites [5] and dengue and chikungunya viruses in *Aedes aegypti* [2], [7], and *Plasmodium* malaria parasites in *Aedes aegypti* [7] and *Anopheles gambiae* [5]. The capacity of *Wolbachia-*infected mosquitoes to transmit these diseases may thus be much reduced.

However, the ability of *Wolbachia* to assist in the control of mosquito-borne diseases will depend on their dynamics in natural mosquito populations. Understanding the ecology of *Wolbachia* infections in mosquito populations is important as programmes to establish *Wolbachia* in wild *Ae. aegypti* are currently under consideration [3]. Recently we developed a modelling framework that allows the spread of *Wolbachia* that reduce the longevity of their insect hosts to be analysed [14]. The models allow the study of the demographic consequences of releasing the significant numbers of individuals often needed to breach the threshold for *Wolbachia* to spread. They can be used to explore different schedules of *Wolbachia* introduction (for example few large or many small introductions of infected insects), the effects of different types of density-dependent mortality in the host population on *Wolbachia* dynamics and the timing of introductions in a seasonal environment.

Here we employ this modelling approach to investigate practical questions concerning the use of *Wolbachia* for mosquito-borne disease management. Because female mosquitoes bite people and so constitute a nuisance, and because they can potentially transmit disease, it is desirable that only a minimum number of female insects are released as part of a *Wolbachia* introduction. This may be possible by applying methods of sex separation by pupal size sorting to reared insects to create releases with a highly male-biased sex-ratio [15], [16]. We develop theory for male-biased release strategies and explore their feasibility and how releases may be optimised when the mosquito population size shows strong seasonal fluctuations. We then extend the model to include a simple representation of a mosquito-borne disease. The model is sufficiently general to represent a wide range of mosquito species and the diseases they transmit; here we chose parameters derived from the literature on *Anopheles* mosquitoes for illustration. This is used to estimate how the rate of disease transmission changes over time following male-biased *Wolbachia* releases. Different assumptions about mosquito population dynamics and the effects of *Wolbachia* on vectorial capacity are explored.

## Methods

### Mosquito and *Wolbachia* dynamics

**Figure 1:**
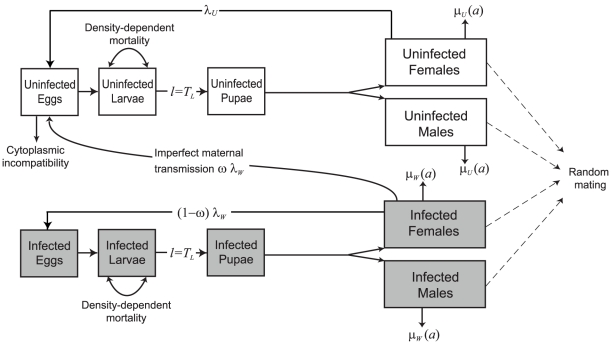
Diagram showing the model structure. Grey-shaded boxes represent mosquito stages infected with *Wolbachia.* Females have fecundity 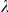, larval development time is *T_L_* days, and adult mosquitoes experience mortality at a rate 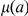 which depends on adult age *a.* Subscripts *W* and *U* denote mosquitoes infected and uninfected with *Wolbachia.* Infected females fail to transmit *Wolbachia* to their offspring with probability 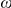.

The model of mosquito and *Wolbachia* dynamics used here is an extension of that in [14] with separate adult sexes and the inclusion of egg and pupal stages (Figure 1). It is phrased as a system of integral equations describing the numbers of infected and uninfected larvae and adults of different ages; full details are given in Text S1.

The mosquito life cycle is divided into three juvenile stages (egg, larva and pupa) and an adult stage. The population is assumed to be regulated by density-dependent mortality experienced during the larval stage described by a power function, 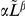, where 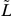 is larval density and 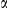 and *β* are constants. Higher values of the parameter *β* denote a steeper response to increasing density (which we shall refer to as strong density dependence). Mortality in adults is assumed to be age-dependent and is modelled by a Weibull function whose parameters may depend on infection status (see Text S3 and [14]). Adult fecundity is assumed to be constant with age (but see the Discussion).

*Wolbachia* may increase adult mortality, particularly in older age-classes, and the proportional reduction in average adult longevity caused by *Wolbachia* is denoted *s_g_*. *Wolbachia-*infected individuals may also have reduced fecundity (by a proportion *s_f_*). Mating is assumed to occur at random, and an uninfected female mating with an infected male will lose a fraction *s_h_* of her offspring. Infected females fail to transmit *Wolbachia* to their offspring with probability 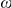. We assume here that *Wolbachia* does not affect survival during, or length of, the juvenile stages.

**Figure 2:**
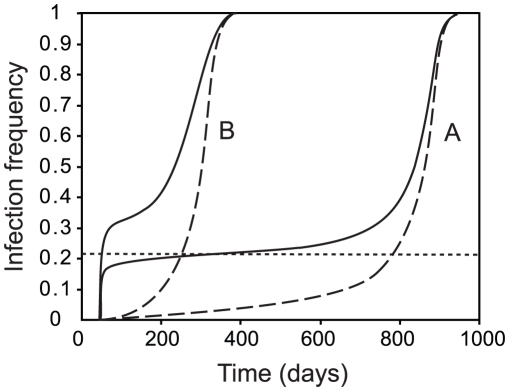
The *Wolbachia* infection frequency in male and female adults following the introduction of infected mosquitoes. Solid lines show the frequency in males and dashed lines show the frequency in females. Introduction is assumed to occur at a constant rate and the rate of female introduction is 1% of the rate of male introduction. Lines A show introduction at the minimum rate required for *Wolbachia* to spread (*I_M_*  =  0.39 day^−1^) and lines B show an introduction rate twice as high as the minimum rate. The dotted line shows the unstable equilibrium male infection frequency. Other parameters are as in Table 1.

For a closed population (no immigration, deliberate introduction, or emigration), the position of the equilibrium threshold frequency above which *Wolbachia* spreads through the population depends on the magnitude of the fitness effects of the bacterium on its host, and the probability of non-transmission (see Figure 2). For the basic model analysed here, Hancock et al. [14] showed that the threshold frequency p*is
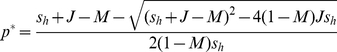
(1)where 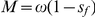 and 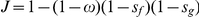. This expression is closely related to the classic condition for spread derived for discrete-generation, purely genetic models by Turelli and Hoffmann [17].

### Seasonal variation in mosquito abundance

**Figure 3:**
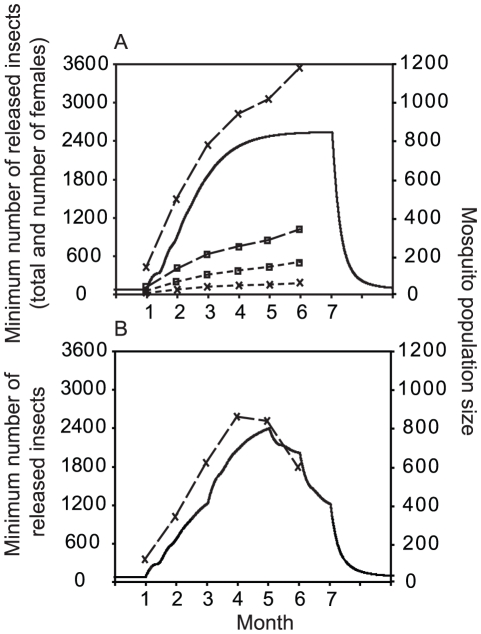
The number of released mosquitoes required for *Wolbachia* to spread at different release times. Solid lines show the mosquito abundance and dashed lines with crosses show the minimum total number of released mosquitoes required for *Wolbachia* to spread. The sex-ratio of the releases is 95% male and 30 equal-sized daily releases are made. Panels A and B show the seasonal mosquito abundance patterns described in the text. Panel A also shows the minimum total number of introduced insects required to cause spread for equal sex-ratio releases (dashed lines, open squares), and the number of females introduced for the 95% male strategy (dotted lines, crosses) and for the equal sex-ratio strategy (dotted lines, open squares). Other parameters are as in Table 1.

Mosquito populations are very sensitive to patterns of seasonal rainfall, and often show strong annual fluctuations in abundance [18]–[20]. We model this by assuming that larval carrying capacity (the parameter 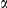 in the expression for larval density dependent mortality) varies over the year. Two seasonal abundance patterns are considered which we refer to as A and B. These patterns were chosen to represent attributes of mosquito population dynamics that we have found to be important to *Wolbachia* spread; strong temporal variation in adult abundance and varying rates of seasonal population growth and decline. In pattern A there is a six-month season of high mosquito abundance generated by setting 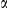  = 0.05 for six consecutive months and 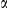  = 0.1 for the rest of the year (Figure 3A; solid line). In pattern B the seasonal increase and decline in mosquito abundance is more gradual (Figure 3B; solid line). This pattern is produced by setting the larval carrying capacity to *α*  =  0.055, 0.055, 0.05, 0.05, 0.053 and 0.06 respectively for the six months of the year when mosquitoes are abundant and 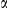  = 0.1 otherwise.

In exploring different release strategies, for operational reasons we restrict deliberate introductions to the wet season. Although the size of the resident population is lowest in the dry season, which would appear to facilitate population replacement, *Anopheles* and other mosquitoes are highly sensitive to desiccation. Mosquitoes may aestivate during the dry season [19], or rest in microhabitats with higher than average humidity. It is likely that if introductions were made at this time of year any introduced mosquitoes would experience very high mortality before locating relatively rare, suitable resting sites (or conspecifics with which to mate).

### Incorporation of disease dynamics

We extend the age-structured model of mosquito and *Wolbachia* dynamics to include a vector-borne pathogen. The infected and uninfected adult classes are divided into susceptible, exposed and infectious (SEI) stages, with the exposed stage assumed to be of fixed duration (the extrinsic incubation period). A fraction *x* of the human population is assumed to be infectious, and this parameter as well as the total number of humans is assumed to be constant over time. The model makes assumptions about the frequency of blood feeding, and the probability of the mosquito being infected during a blood meal. Our treatment of the adult stages is based on Hancock et al. [21] (a model of the interaction between *Anopheles, Plasmodium* and a pathogenic fungus). Full details of the model are given in Text S2. We assume here that a proportion *c_w_* of mosquitoes that are infected with both *Wolbachia* and the pathogen do not become infectious. This represents the reduction in disease transmission that has been shown to occur in mosquitoes infected with *Wolbachia*.

A critical quantity in the epidemiology of mosquito-borne diseases is the Entomological Inoculation Rate (EIR), the number of bites on humans by infectious mosquitoes per person per day. This is simply the total number of infectious mosquitoes (both carrying and not carrying *Wolbachia*) per human multiplied by the daily rate of biting [22]. Analytical expressions for the equilibrium EIR can be obtained for a constant environment where *Wolbachia* is absent or at equilibrium frequencies (Text S2).

### Model parameterisation

The different parameters included in the models and their default values are shown in Table 1. Parameter estimates obtained in the field for *Anopheles* mosquitoes have been used where possible, though for some such as those governing density dependent mortality little information is available. Data on age-dependent mortality rates of laboratory colonies of *Anopheles* were used to parameterise the Weibull function describing adult age-dependent mortality [23]. We assume that mosquitoes experience additional age-independent background mortality at rates observed in field populations (see Text S3).

**Table 1:** The parameters used in the model and their default values.

Symbol	Definition	Value	Source
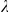	Daily female fecundity for mosquitoes uninfected with *Wolbachia*	30	[30]
*T_0_*, *T_L_*, *T_P_*	Development time in days of eggs, larvae and pupae	1, 10, 1	[30]
*µ_0_*, *µ_P_*	Daily mortality rate of eggs and pupae	0.1	[31], [30]
*µ, α, β*	Parameters describing the density-dependent larval mortality function	0.1,0.1, 0.2	No data available
*c*,*γ, r* *c_w_*, *γ_w_, r_w_*	Parameters describing the age-dependent adult mortality function for mosquitoes uninfected and infected with *Wolbachia*	0.1, 0.025, 2.00.1, 0.05, 2.0	[23], [6], [30]
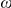	Fraction of uninfected larvae produced by an infected adult female	0.01	[6]
*s_h_*	Fraction of eggs that fail to hatch from an incompatible mating	0.99	[6]
*s_f_*	Proportional reduction in fecundity due to *Wolbachia* carriage	0.05	[6]
*s_g_*	Proportional reduction in average adult lifespan due to *Wolbachia*	0.16	[6]
*f*	Daily human biting rate	0.3	[30]
*c*	Pathogen transmission efficiency from mosquitoes to humans for mosquitoes and vice versa	0.5	[32], [33]
*c_w_*	Proportion of mosquitoes infected with *Wolbachia* and pathogen that do not become infectious	varies	[7]
*x*	Proportion of infectious humans	0.5	No data available
*T_E_*	Pathogen incubation period in days	10	[34]
*H*	Number of humans	120	No data available

Parameters describing the effect of *Wolbachia* on longevity derive from field cage studies of the life-shortening *Wolbachia* strain *w*MelPop infecting *Ae. aegypti* [6]. We calculated the age-dependent increase in the rate of adult mortality caused by *w*MelPop infection in *Ae. aegypti* and assumed that it would have a similar proportional effect on *Anopheles* (Text S3). Mosquitoes in cages tend to live longer than those in nature and this can lead to overestimation of the fitness consequences of late-acting mortality. Including background field mortality, the overall reduction in average adult lifespan caused by *Wolbachia* infection is assumed to be 16% (*s_g_* = 0.16) (Text S3). Both strains of *Wolbachia* that have to date been successfully introduced into *Ae. aegypti*, *w*MelPop and *w*AlbB, inhibit the development and transmission of human pathogens in this host [2], [4], [7], and unlike *w*MelPop the *w*AlbB transinfection had no observable impact on longevity in the lab [2]. We also explore the effects of introducing a *Wolbachia* that causes a 5% reduction in adult lifespan in the field (*s_g_* = 0.05). We address the implications of our imperfect knowledge of different parameters in the Results and Discussion.

## Results

### Equilibrium results for highly male-biased releases

When transmission is perfect (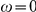), *Wolbachia* spreads when it reaches an infection frequency in the population such that an average infected female has more offspring than an average uninfected female. The latter are disadvantaged through mating with infected males which causes them to lose a fraction *s_h_* of their offspring. This picture is slightly more complex when transmission is not perfect, or when immigration, introductions or emigration are occurring [14], but again spread is caused by the presence of infected males giving an indirect, relative advantage to *Wolbachia-*bearing females. This advantage can be made greater simply by increasing male (and not female) infection frequency. Of course infected females must be present for the infection (which is not transmitted through males) to spread, but once the threshold is exceeded the frequency in females will increase from an arbitrarily low start. An infection can thus be established even though relatively few females are released.

A simple way to model sex-biased releases is to assume that newly-emerged infected males and females are introduced into an uninfected population at constant rates *I_M_* and *I_F_*. For simplicity we assume that the larval carrying capacity does not vary with time (no seasonality) and that the uninfected population is at equilibrium prior to the introduction. Figure 2 illustrates how introducing males at a relatively high rate allows the infection to invade when females are introduced at a much lower rate (1% of the rate of male introduction). The time it takes for the infection to be established is longer when introduction rates are low.

When the rate at which infected females are introduced is very small (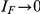), it is possible to calculate the unstable equilibrium male infection frequency above which *Wolbachia* spreads, and the threshold rate of male introduction required to exceed this frequency (Figure 2 and Text S1). However the expressions are complicated because the introduction of infected males reduces the fecundity of resident uninfected females and this lowers the density dependent mortality experienced by the juvenile population. The effect of the introduction on the rate of recruitment of uninfected adults will thus depend on the strength of juvenile density dependence. This is illustrated by comparing the threshold male introduction rates required for *Wolbachia* spread to occur in populations with relatively strong (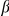  = 0.3, 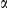  = 0.05) and weak density dependence (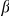  = 0.1, 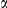  = 0.2). Values of the larval carrying capacity 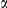 were chosen so that the equilibrium adult abundance in the absence of *Wolbachia* is the same in both cases. The required rate of male introduction is approximately 50% higher in the case of strong (*I_M_*  =  0.44 day^−1^) as opposed to the weak (*I_M_*  = 0.28 day^−1^) density dependence. This occurs because the reduction in density-dependent mortality caused by the introduction of males is greater when density dependence is stronger, and so less suppression of the (uninfected) adult population occurs. It can thus be important to consider demographic as well as genetic processes in models of *Wolbachia* dynamics.

### Highly male-biased releases in separate batches

A more realistic scenario for the release of *Wolbachia*-infected mosquitoes is that the insects are released in separate batches rather than continuously, and that mosquito population size fluctuates seasonally. The total and relative numbers of male and female mosquitoes that need to be released for spread to occur were studied in a population whose seasonal dynamics are described by pattern A. We compare releases consisting of equal numbers of the two sexes and 95% males and calculate the minimum numbers that have to be liberated at different times of the season to ensure *Wolbachia* becomes established. The release strategy we model is of 30 daily releases, each containing the same number of mosquitoes.

The results are shown in Figure 3A. First note that for all strategies releases early in the season when the resident population is small require fewer mosquitoes to be introduced, a result we explore in more detail below. Overall, for any particular release date, the total required release size is 3–4 times larger for the 95% male-biased strategy compared to the equal sex-ratio strategy, and so the number of mosquitoes that must be reared (prior to separation of the sexes) assuming a 50∶50 sex-ratio is 6–8 times greater. However, although more mosquitoes in total must be produced, fewer females need to be released with the male-biased strategy. In the present example, which is typical of others we have explored, the total number of females introduced is approximately ⅓–½ the numbers required in the equal sex-ratio strategy.

**Figure 4:**
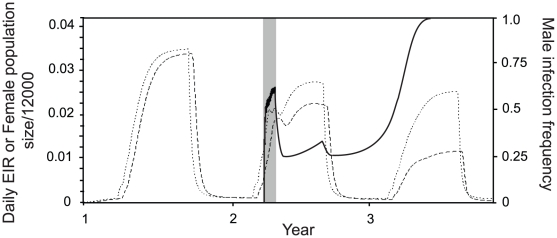
The daily EIR (dashed line) and female population size (dotted line) following male-biased *Wolbachia* releases. A strategy of 30 daily 95% male releases of the minimum number of *Wolbachia-*infected mosquitoes required for spread is shown. Releases begin in the second year in the second month of the high-abundance season. Seasonal dynamics follow pattern A. The solid line shows the male infection frequency and the shaded area shows the period during which releases occur. Other parameters are as in Table 1.

There are two reasons why the male-biased strategy requires the release of fewer females. First, releasing a large number of males causes a high frequency of incompatible matings and so reduces the size of the resident (uninfected) population. Second, the high frequency of infected males means that infected females have a strong relative fitness advantage. However, the dynamics of releasing mosquitoes in a finite number of separate batches are not the same as those assuming introduction at a constant rate. Figure 4 shows the male infection frequency as a function of time over a 3 year period following 30 daily 95% male releases made in the second month of the season of high mosquito abundance. Although male infection frequencies are initially very high they decline rapidly after the final release as the introduced males die. At this stage there are still relatively few infected females present and hence recruitment of *Wolbachia*-carrying individuals is low. To prevent the loss of *Wolbachia* in this transient period, the releases must attain a temporary male infection frequency that is considerably higher than the threshold calculated in the continuous release case. Enough females must also be introduced so that they produce sufficient infected sons that the male infection frequency does not fall below the threshold following the final release.

As in the case of continuous release, we found that the minimum required number of insects for *Wolbachia* establishment depended on the assumed form of juvenile density-dependent mortality. Further details are given in Text S4 but we again found that larger releases were needed when density dependence was strong. However, for all the forms of density dependent mortality we studied, consistently ⅓–½ the number of females was required for male-biased (95%) compared to equal sex-ratio releases.

These results suggest that the establishment of *Wolbachia* using highly male-biased releases is feasible, provided comparatively large numbers of mosquitoes can be reared and the sexes separated. We explore this issue further in the Discussion.

### Factors affecting the required release size: time during the season

We explored the effect of seasonal variation in mosquito abundance on the release size necessary for *Wolbachia* to spread for the two seasonal patterns described in the Model Development. Again we assume that 95% of the insects introduced are males, and that 30 daily releases of the same size are made. Figures 3A & B show the minimum required release size for different release times.

In both cases releases early in the season require fewer mosquitoes. At this time the resident population is small and so a fixed number of introduced infected insects constitute a greater proportion of the total. Releases made towards the end of the season when the population is beginning to decrease may also require fewer insects, but this depends on the rate of population decline. In case A the decline is abrupt and the size of releases required for spread increases steadily through the season. Late season releases here are a poor strategy because the decline in larval carrying capacity drastically reduces recruitment to the adult stage so that the proportion of infected individuals is chiefly determined by adult mortality rates that with our parameter assumptions particularly penalise *Wolbachia*-carrying individuals. However, in case B, where the population declines more gradually, the required release size decreases towards the end of the season.

Insight into the seasonal dynamics of *Wolbachia* spread following the releases can be gained by plotting the temporal change in male infection frequency for seasonal pattern A (Figure 4). The male infection frequency falls following the final release and then starts to rise again towards the end of the season as the progeny of the first female introductions reach the adult stage. However, the collapse in carrying capacity acts to reduce the infection frequency as the season ends, for the reasons described above. *Wolbachia* only becomes established if the infection frequency towards the end of the season is high enough that it does not drop below the threshold (eqn. 1) when the population enters the steep decline. This is a further reason why *Wolbachia* strains that reduce host longevity require large releases before they can become established.

### Factors affecting the required release size: number of releases

Models of the introduction of *Wolbachia* with equal numbers of males and females show that the number of releases made can significantly affect the total number of insects that need to be introduced to establish *Wolbachia* in the population [14]. In particular, introducing large numbers of females at one time that then reproduce can increase the juvenile density-dependent mortality, which disadvantages the progeny of these females. Introducing the insects in a larger number of smaller releases is therefore sometimes more effective, particularly for *Wolbachia* strains that incur a high fitness cost and thus require large releases to allow spread.

In the case of highly male-biased releases, this effect does not occur, because relatively few females are added and the number of larvae declines due to the high frequency of incompatible matings. However multiple releases may still be beneficial because they prolong the period over which the male infection frequency is artificially elevated, so sustaining the fitness advantage of infected females and allowing their numbers to increase from an initial low level.

For seasonal pattern A, we compared the minimum number of introduced insects required for spread for strategies where different numbers of equal-sized, daily releases are made, again assuming that the sex-ratio of the releases is 95% male (see Text S5). If releases are made towards the middle of the season, after the period of rapid population growth, the total required number of introduced insects is smaller if the insects are distributed across a larger number of batches. This is not the case for releases made close to the start of the season, when there is a slight advantage in releasing the mosquitoes in a single batch. At the start of the season the benefit of prolonging the elevation of the male infection frequency over multiple releases is lost because the population is increasing rapidly and so later releases cause a smaller increase in the proportion infected. These results indicate that seasonal changes in mosquito abundance are a much stronger determinant of the required release size than the number of releases made (Text S5).

### Male-biased releases, female numbers and the EIR

Here we examine the effects of *Wolbachia* introduction on the abundance of female mosquitoes and the rate of disease transmission for a release strategy that introduces the minimum number of mosquitoes required for spread in 30 daily equal-sized batches, and a strategy that releases a larger number, three times the minimum required, in 90 daily equal-sized batches. The sex-ratio of the releases is 95% male. We assume that seasonal abundance dynamics follow pattern A, and that releases are made one month into the season of high mosquito abundance.

Figure 4 shows the daily entomological inoculation rate (EIR), the female population size, and the male infection frequency for a three year period where releases of the minimum size required for spread are made in the second year. During the releases the total number of females (including wild and released individuals), and likewise the EIR, are quickly reduced compared to the level in the previous year, although at the start of the releases there are slightly more females present than there would be in the absence of the intervention. The reduction in both quantities is due mainly to the suppression in population abundance caused by the high frequency of incompatible matings between uninfected females and infected males. The reduction also partly results from the lower fitness of *Wolbachia-*infected females, although this does not have a strong effect in the year of release because the *Wolbachia* infection frequency in females remains relatively low. In this example the *Wolbachia* does not reach its stable frequency until the year following the releases and its establishment results in much greater reduction in EIR than in the female population size (Figure 4). This is because the reduction in longevity brought about by *Wolbachia* infection causes a disproportionate reduction in the abundance of individuals that live long enough to transmit the pathogen.

**Figure 5:**
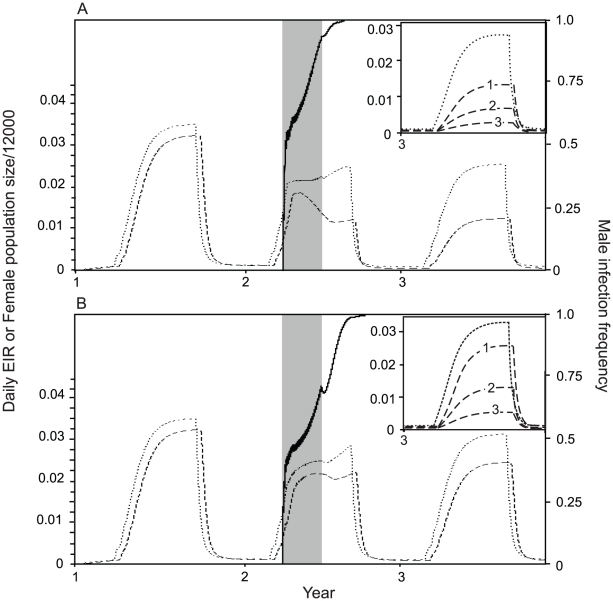
Daily EIR (dashed line) and female population size (dotted line) following extended *Wolbachia* releases. A strategy of 90 daily releases of 95% male, *Wolbachia-*infected mosquitoes is shown. The solid line shows the male infection frequency and the shaded area shows the period during which releases occur. Panels show different effects of *Wolbachia* on adult longevity: A. a 16% reduction in average adult lifespan (*s_g_* = 0.16), B. a 5% reduction in average adult lifespan (*s_g_* = 0.05, *r_w_* = 0.03). Insets show the same results for the third year only, and also include lines showing the EIR for different effects of *Wolbachia* on pathogen development; lines labelled 1,2 and 3 show *c_w_* = 0, *c_w_* = 0.5 and *c_w_* = 0.8 respectively. Other parameters are as in Table 1.

We now compare these dynamics to those produced when the total number of insects released is three times the minimum required (Figure 5A). In this case the *Wolbachia* spreads much more rapidly and reaches its final frequency in the year of release. Although more insects are introduced there is still only a very slight initial increase in the female population size at the start of releases, followed rapidly by a net reduction in both population size and EIR. An advantage of releasing more mosquitoes is that the EIR declines more quickly due to faster *Wolbachia* spread.

In addition to reducing adult longevity, *Wolbachia* can also directly inhibit pathogens within the mosquito. The insets in Figure 5 show the combined effects of life-shortening and pathogen inhibition on the EIR once *Wolbachia* has become established. We assume that *Wolbachia* reduces average adult longevity by either 16% (Figure 5A) or 5% (Figure 5B). Reducing longevity has a major impact on the EIR but in both cases direct pathogen inhibition gives a further substantial decrease in the EIR. Our results show that a 16% reduction in longevity with no effect on transmission is similar to the joint effect of a 5% reduction in lifespan with a 50% reduction in transmission. However, it would require far fewer mosquitoes to be released to establish a *Wolbachia* with the second phenotype.

We explored how these conclusions were affected by the nature of the assumed density-dependence (see Text S6). When density-dependence is strong the releases cause less reduction in the female population size, both transiently due to incompatible matings and in the long term due to the fitness costs of *Wolbachia* infection. However the reduction in the EIR was similar for all the forms of density dependence we considered, particularly in the longer term once the *Wolbachia* has reached a high infection frequency. This is because the EIR is much more sensitive to changes in adult mortality than to changes in the rate of adult recruitment.

## Discussion

Introducing *Wolbachia* into mosquito populations can lead to a reduction in the transmission of mosquito-borne diseases. The normal way in which establishment has been envisioned is through the release of equal numbers of male and female mosquitoes [3]. However, as females transmit disease and are responsible for nuisance biting, it is important to minimise the numbers of females released, and this may be critical in obtaining regulatory permission and public support for introductions. It is shown here that establishment can occur following releases composed very largely of males provided this causes the *Wolbachia* infection frequency in males in the field to exceed a threshold. The numbers of females in the population decline rapidly following the initial male-biased releases, and only for a relatively brief period at the commencement of releases are female numbers slightly higher than they would have been in the absence of the intervention. However, for male-biased releases the numbers of insects that must be reared is considerably higher than when releases are composed of equal numbers of the two sexes, and a reliable method must exist for separating males and females. These may not be major barriers to the strategy. Some mass-rearing facilities have the capacity to produce over 1 million mosquitoes per week [15], which is more than 30 times the estimated size of some village-scale natural mosquito populations [3], [24]. For *Aedes aegypti*, male and female pupae can be rapidly separated by size and when larval rearing conditions are optimal over 99% males can be achieved [15], [16]. Transgenic sex separation methodologies also exist (e.g. Alphey et al. [15]), although their use would lose the advantages of *Wolbachia* intervention not involving genetically modified organisms.

However in situations where the number of insects that can be reared and released is more strongly limited, such as in isolated rural areas, it may be preferable to use equal sex-ratio releases which will provide more rapid *Wolbachia* spread for a given available release size and number. This would improve the likelihood of achieving *Wolbachia* establishment, for example in the case of unexpected losses of released insects. Equal sex-ratio releases result in considerably less suppression of female numbers as well as of the EIR during the release period compared to male-biased releases. They also lead to higher densities of biting females, though the increase over natural levels is not always very marked (see Text S7). Release programmes that have the capacity for bigger release sizes and greater numbers of releases are likely to gain stronger benefit from using male-biased releases to limit the addition of females and suppress the vector population. Engagement with local communities will also reveal the extent to which the possibility of modest and temporary increases in biting female numbers would be a significant impediment to their support for the program.

A further strategy for reducing the risk of increased biting or disease transmission associated with the introduction of females is artificially to suppress the mosquito population prior to release, for example by insecticidal fogging or larval control. Fewer released mosquitoes would then be required to surpass the threshold infection frequency that allows *Wolbachia* to spread. Suppression measures would be stopped immediately prior to mosquito release so as to minimise the time for population numbers to rebound, and so as not to affect the introduced insects. The efficacy of different types of pre-release suppression will depend on the population dynamics of the mosquito species, in particular the form of density dependence, and can be explored using the type of model developed here (see Text S8 for examples).

Once *Wolbachia* becomes established in the population, the model indicates that the rate of disease transmission can be substantially reduced due to the bacteria both reducing adult mosquito longevity and inhibiting pathogen transmission. The pathogen inhibition phenotype of *Wolbachia* described for the *w*MelPop strain in *Ae. aegypti* [5], [7]and *An. gambiae* [4] is also produced by some, but not all, other strains of the bacterium, both in *Drosophila* [25]–[27] and *Ae. aegypti* [2]. To date only *w*MelPop has been shown to produce a significant reduction in lifespan, but it seems reasonable to predict that other strains will also produce some degree of life shortening when moved into a naïve mosquito host, particularly if costly immune pathways are activated [4], [5], [7]. Our results indicate that even a small reduction in adult longevity acts together with the direct effects of *Wolbachia* on the pathogen to produce a considerably greater reduction in pathogen transmission.

In general *Wolbachia* strains that induce strong pathogen inhibition with minimal or no associated life-shortening would be the optimal choice for use in disease control strategies, since this would reduce the level of releases that are required, improve spread dynamics, and minimize selective pressure for modulation of phenotypes that reduce pathogen transmission. Whether the additional *Wolbachia*-associated mortality occurs only in late life or throughout adulthood will also determine the strength of the selective pressure for modulation of the phenotype, and thus how long-lasting the strategy is likely to be in providing disease control. Sub-lethal effects could for example affect the capacity of young adults to escape predation in the wild. Ultimately it may only prove possible to obtain a full understanding of how cage survival dynamics translate to natural conditions, and relative mortalities in captive-bred versus wild insects, once field releases are actually underway.

Other aspects of mosquito biology that have not been considered here may also be important to *Wolbachia* spread dynamics. For example, in *Aedes aegypti* old females have been shown to have lower fecundity [28], [29], and this may reduce the fitness costs of *Wolbachia* infection if its effects on the host are strongest late in life. An extended version of the model that incorporates age-dependent fecundity can be analysed using the methods presented in Text S1. However we currently know little about the interactions between mosquito age and the effects of *Wolbachia* infection on fecundity for any mosquito species. This emphasises the need for detailed empirical study of the effects of *Wolbachia* on mosquito demography.

In conclusion, our results show that the establishment of *Wolbachia* in natural mosquito populations using male-biased releases is feasible provided that the mass-rearing capacity is available for the larger number of insects that need to be reared. Successful establishment of *Wolbachia* strains which reduce mosquito longevity or interfere with the pathogen in its vector are predicted to have substantial long-term benefits in terms of reduced disease transmission, and employing male-biased introductions minimises the risk of any biting or disease transmission during the release period.

## Supporting Information

Text S1 Sex-structured model of mosquito and *Wolbachia* dynamics and equilibria.
(PDF)

Text S2 Modelling mosquito-borne disease transmission.
(PDF)

Text S3 Modelling adult age-dependent mortality.
(PDF)

Text S4 The effect of juvenile density-dependent mortality on the required number of released females.
(PDF)

Text S5 The effect of varying the number of releases on the required release size.
(PDF)

Text S6 The effect of male-biased *Wolbachia* release on female population size and EIR for different forms of juvenile density-dependence.
(PDF)

Text S7 Effects of equal sex ratio releases on the EIR and female population size.
(PDF)

Text S8 *Wolbachia* introduction following pre-release population suppression.
(PDF)
