# Supplementary material for: Strategies for Introducing Wolbachia to Reduce Transmission of Mosquito-Borne Diseases
Source: PLoS Negl Trop Dis. 2011 Apr 26;5(4):e1024. doi: 10.1371/journal.pntd.0001024 (PMC3082501; doi:10.1371/journal.pntd.0001024)
Supplement: Text S1 — Sex-structured model of mosquito and Wolbachia dynamics and equilibria. (PDF) [file pntd.0001024.s001.pdf]

Strategies for introducing *Wolbachia* to reduce transmission of mosquito-borne diseases

Penelope A. Hancock, Steven P. Sinkins & H. Charles J. Godfray

Text S1: Sex-structured model of mosquito and *Wolbachia* dynamics and equilibria

Mosquito population dynamics are modelled using a similar approach to Hancock et al. [14], which describes the dynamics of mosquito larva and adults of different ages using a system of integral equations. Here we extend the model to separate the male and female sexes, and include egg and pupal juvenile development stages.

The mosquito population is divided into classes of juveniles and adults. The juvenile population is divided into classes of eggs, larvae and pupae and the adult population is divided into classes of females and males. The dynamics of larvae and adults are modelled explicitly. Define $L(t,l)$ to be the numbers of larvae at time t which have been in the larval stage for time l. Let the probability a larva survives until time l be $\theta_L(t,l)$ which because larval mortality may be density dependent is a function of time. The total time spent in juvenile development is the sum of the maximum durations of the egg, larval and pupal stages, T_O, T_L and T_P. Egg and pupal mortality are assumed to be density-independent, and total egg and pupal survival are defined as θ_O and θ_P.

Define $A(t,a)$ to be the total number of adults of age a at time t. The probability an adult survives until age a is defined as $\theta_A(a)$ which depends on age alone. Let $\tilde{L}(t)$ and $\tilde{A}(t)$ be the total number of larvae and adults at time t (obtained by integrating over all age classes) and define the female fecundity per unit of time, λ.

Mosquitoes uninfected and infected with *Wolbachia* are represented by subscripts U and W. At any one time a proportion $p_M(t)$ of male adults are infected by *Wolbachia* and a fraction s_h of the offspring of any uninfected female that mates with an infected male will fail to develop. Mating is assumed to occur at random. Infected females fail to transmit *Wolbachia* to a fraction ω of their offspring (which if fertilised by sperm from uninfected males fail to hatch with probability s_h). In accordance with empirical observations of *Wolbachia* infection in mosquito populations we will consider that *Wolbachia* may affect adult mortality, possibly in age-dependent manner, and female fecundity. However for simplicity we assume that *Wolbachia* infection does not affect the mortality or the development time of eggs, larvae and pupae.

We write separate systems of equations for the dynamics of male and female adults, representing males and females by subscripts M and F. The dynamics of adult females are described by

$$L_W(t,l) = \lambda_W (1-\omega)\tilde{A}_{WF}(t-T_O-l)\theta_O\theta_L(t,l), \qquad l \le T_L$$
$$L_U(t,l) = (1-s_h p_M(t-T_O-l))\left[\lambda_U \tilde{A}_{UF}(t-T_O-l) + \omega\lambda_W \tilde{A}_{WF}(t-T_O-l)\right]\theta_O\theta_L(t,l), \quad l \le T_L$$
$$A_{WF}(t,a) = \frac{1}{2}L_W(t-T_P-a,T_L)\theta_P\theta_{A,W}(a)$$
$$A_{UF}(t,a) = \frac{1}{2}L_U(t-T_P-a,T_L)\theta_P\theta_{A,U}(a) \qquad \text{(A1 a-d)}$$

The dynamics of adult males are given by

$$A_{WM}(t,a) = \frac{1}{2}L_W(t-T_P-a,T_L)\theta_P\theta_{A,W}(a)$$
$$A_{UM}(t,a) = \frac{1}{2}L_U(t-T_P-a,T_U)\theta_P\theta_{A,U}(a) \qquad \text{(A2 a-b)}$$

where the numbers of infected and uninfected larvae are also described by eqns A1a & b. In modelling sex-biased introduction of *Wolbachia*-infected insects we assume that male and female *Wolbachia*-infected mosquitoes are introduced into an uninfected population at rates $I_M(t)$ and $I_F(t)$. Equations (A1c) and (A2a) are therefore modified to give

$$A_{WF}(t,a) = (\frac{1}{2}L_W(t-T_P-a,T_L)\theta_P + I_F(t))\theta_{A,W}(a) \qquad \text{(A3)}$$

$$A_{WM}(t,a) = (\frac{1}{2}L_W(t-T_P-a,T_L)\theta_P + I_M(t))\theta_{A,W}(a) \qquad \text{(A4)}$$

Male infection frequency equilibria for constant male introduction

We solve for the stable and unstable male infection frequency equilibria, p_M^*, for the limiting case in which the rate of introduction of infected females is negligible, $I_F(t) \to 0$ and the rate of infected male introduction is a constant I_M. At the equilibrium, denoted by asterisks, systems (A1-A4) are written as

$$L_W^*(l) = \frac{\lambda_W}{2}(1-\omega)\tilde{A}_{WF}^*\theta_O\theta_L^*(l), \qquad l \le T_L$$
$$L_U^*(l) = (1-s_h p_M^*)\left[\frac{\lambda_U}{2}\tilde{A}_{UF}^* + \omega\frac{\lambda_W}{2}\tilde{A}_{WF}^*\right]\theta_O\theta_L^*(l), \quad l \le T_L$$
$$\tilde{A}_{WF}^* = L_W^*(T_L)\theta_P\int_0^\infty \theta_{S,W}(a)da = L_W^*(T_L)\theta_P\Theta_W \qquad \text{(A5a-d)}$$
$$\tilde{A}_{UF}^* = L_U^*(T_L)\theta_P\int_0^\infty \theta_{S,U}(a)da = L_U^*(T_L)\theta_P\Theta_U$$

and

$$\tilde{A}^*_{WM} = (L^*_W(T_L)\theta_P + I_M)\int_0^\infty \theta_{S,W}(a)da = (L^*_W(T_L)\theta_P + I_M)\Theta_W$$
$$\tilde{A}^*_{UM} = L^*_U(T_L)\theta_P\int_0^\infty \theta_{S,U}(a)da = L^*_U(T_L)\theta_P\Theta_U$$

where the average lifespans of adults infected and uninfected with *Wolbachia* are Θ_W and Θ_U respectively.

Substitute A1a in A1c to give

$$\tilde{A}^*_{WF} = \frac{\lambda_W}{2}(1-\omega)\tilde{A}^*_{WF}\theta^*_L(T_L)\theta_O\theta_P\Theta_W$$
$$\Rightarrow \theta^*_L(T_L) = \frac{2}{\lambda_W(1-\omega)\theta_O\theta_P\Theta_W} \quad \text{iff} \quad \tilde{A}^*_{WF} \neq 0 \tag{A6}$$

Substituting A1b into A1d gives

$$\tilde{A}^*_{UF} = (1-s_h p^*_M)\left[\frac{\lambda_U}{2}\tilde{A}^*_{UF} + \omega\frac{\lambda_W}{2}\tilde{A}^*_{WF}\right]\theta^*_L(T_L)\theta_O\theta_P\Theta_U\,. \tag{A7}$$

Substitute (A6) into (A7) to give

$$\tilde{A}^*_{UF} = (1-s_h p^*_M)\left[\lambda_U\tilde{A}^*_{UF} + \omega\lambda_W\tilde{A}^*_{WF}\right]\frac{\Theta_U}{\lambda_W(1-\omega)\Theta_W}\,. \tag{A8}$$

Expressing this in terms of equilibrium adult male abundances gives

$$\tilde{A}^*_{UM} = (1-s_h p^*_M)\left[\frac{\lambda_U}{2}\tilde{A}^*_{UM} + \omega\frac{\lambda_W}{2}(\tilde{A}^*_{WM} - I_M\Theta_W)\right]\frac{\Theta_U}{\lambda_W(1-\omega)\Theta_W} \tag{A9}$$

Make the substitution

$$I_M = \varepsilon\frac{A^*_{WM}}{\Theta_W} \tag{A10}$$

where $0 \leq \varepsilon \leq 1$, and divide by $\tilde{A}^*_{UM} + \tilde{A}^*_{WM}$ to give

$$p_M^* = (1 - s_h p_M^*)\left[\frac{\lambda_U}{2} p_M^* + \omega \frac{\lambda_W}{2}(1-\varepsilon) p_M^*\right] \frac{\Theta_U}{\lambda_W (1-\omega)\Theta_W} \qquad \text{(A11)}$$

Let $\lambda_W = (1 - s_f)\lambda_U$, $\Theta_W = (1 - s_g)\Theta_U$; and simplify to give the quadratic

$$(1 - p_M^*) = \frac{(1 - s_h p_M^*)[(1 - p_M^*) + \omega(1-\varepsilon)(1 - s_f) p_M^*]}{(1 - s_f)(1 - s_g)(1 - \omega(1-\varepsilon))}, \qquad \text{(A12)}$$

The lower unstable and upper stable equilibrium male infection frequencies are given by

$$p_M^* = \frac{s_h + J - M \pm \sqrt{(s_h + J - M)^2 - 4(1-M)Js_h}}{2(1-M)s_h} \qquad \text{(A13)}$$

where $M = \omega(1-\varepsilon)(1-s_f)$ and $J = 1 - (1 - \omega(1-\varepsilon))(1-s_f)(1-s_g)$. It can be seen from this expression that if the rate of male introduction I_M is zero, the infection frequency equilibria are the same as those obtained when an equal sex-ratio is assumed (eqn 1). If transmission of *Wolbachia* is perfect (ω =0) then the steady introduction of males makes no difference to the equilibria.

In the more general case ($\omega \geq 0$) it is possible to solve for the unstable equilibrium male infection frequency. From Hancock et al. (*in press*) we know that the equilibrium abundance of infected females is given by

$$\tilde{A}_{WF}^* = \frac{2 L_T^* p_M^*}{\theta_L^*(\lambda_U (1 - s_h p_M^*)(1 - p_M^*) + \lambda_W \omega p_M^* (1 - s_h p_M^*) + \lambda_W (1-\omega) p_M^*)} \qquad \text{(A14)}$$

where the equilibrium total density of larvae, $\tilde{L}_T^* = \tilde{L}_W^* + \tilde{L}_U^*$, is given by (A6). Also note that $A_{WM}^* = A_{WF}^* + I_M \Theta_W$. Values of the unstable equilibrium $\tilde{A}_{WM}^*$ as a function of ε are given by substituting the lower value of the expression for p_M^* (eqn A13) and the expression for the total larval density $\tilde{L}_T^*$ eqn (A6) into eqn (A14). The male introduction rate I_M as a function of ε is then given by eqn (A10). Figure S1 shows the equilibrium p_M^* for different values of I_M for a relatively low rate of maternal transmission of *Wolbachia* (ω =0.3). The threshold frequency declines as the rate of male introduction increases, asymptoting to the value obtained when maternal transmission is perfect (ω =0). This occurs because the introduction of infected males reduces the rate of recruitment of uninfected individuals to the adult population by increasing the frequency of incompatible matings. This counteracts the

additional production of uninfected offspring by infected females due to imperfect maternal transmission of *Wolbachia.*

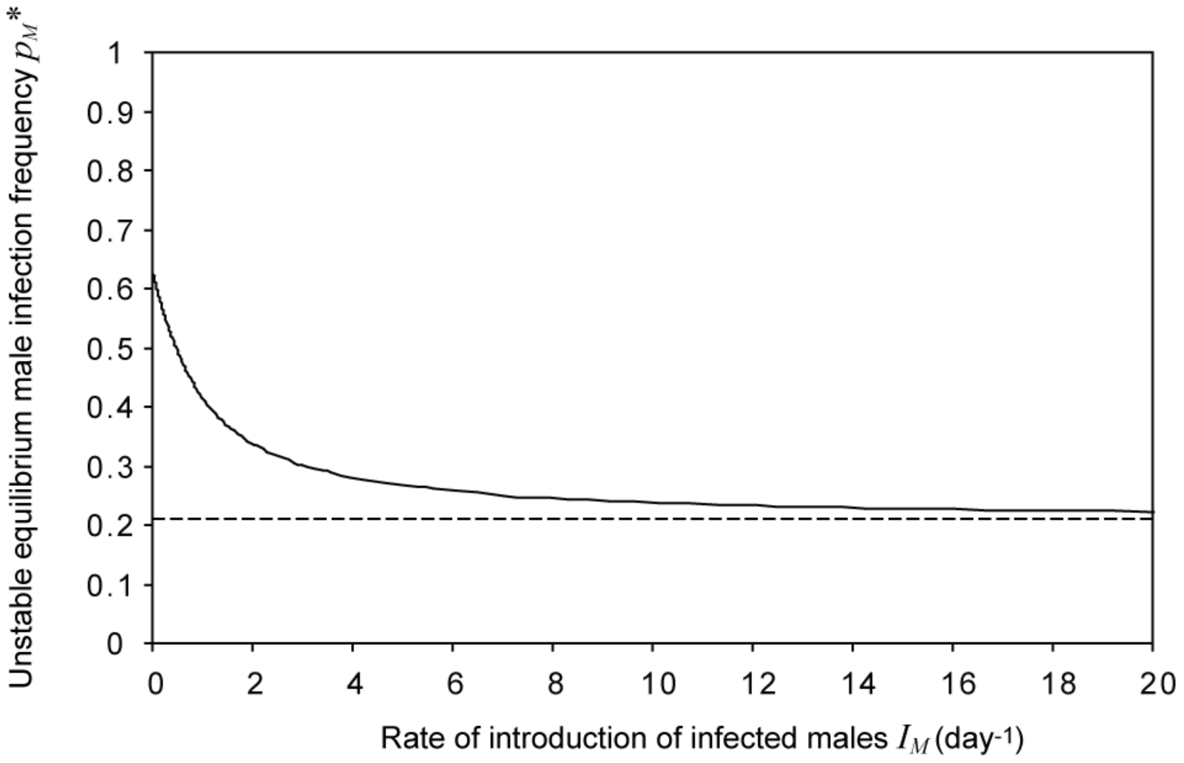

Figure S1. The unstable equilibrium male infection frequency as a function of the rate at which infected males are introduced. The probability that an offspring of a *Wolbachia*-infected mother is uninfected is ω =0.3 and other parameters are as in Table 1. The dashed line shows the unstable equilibrium male infection frequency when maternal transmission of *Wolbachia* is perfect, ω =0.

The threshold rate of male introduction required for Wolbachia spread

The threshold rate of male introduction required for *Wolbachia* to spread is given by the value of I_M that attains a male infection frequency equal to the unstable equilibrium. To solve for this threshold, we now consider that there are no infected females at equilibrium, $\tilde{A}^*_{WF} = 0$. Now from eqns A5b & d we have

$$\tilde{A}^*_{UF} = \frac{\lambda_U}{2}(1 - s_h p^*_{MI})\tilde{A}^*_{UF}\theta^*_L(T_L)\theta_O\theta_P\Theta_U \qquad \text{(A15)}$$

where p^*_{MI} is the equilibrium male infection frequency attained by the male introduction at rate I_M. This gives

$$p_{MI}^* = \frac{\lambda_U \theta_L^*(T_L)\theta_O \theta_P \Theta_U - 2}{s_h \lambda_U \theta_L^*(T_L)\theta_O \theta_P \Theta_U} \qquad \text{(A16)}$$

Therefore the male infection frequency attained depends on the larval survival at equilibrium, $\theta_L^*(T_L)$, which is a function of the equilibrium larval density. The reduction in larval density caused by the introduction of males depends on the form of density dependent mortality, and therefore the increase in male infection frequency caused by a given rate of introduction will also vary with the form of density-dependence. To find the threshold introduction rate required for *Wolbachia* to spread we need to solve for the value of I_M for which p_{MI}^* (eqn A16) is equal to the unstable equilibrium p_M^* (eqn A13). This can be solved using numerical solution methods.

Figure S2 shows how the form of density dependence affects the threshold introduction rate required for *Wolbachia* to spread for host populations which all have the same equilibrium adult abundance in the absence of *Wolbachia*. Higher rates of infected male introduction are required when density-dependence is strong. This is because the reduction in density-dependent mortality caused by the introduction of males is greater when density-dependence is stronger, and so less suppression of the (uninfected) adult population occurs.

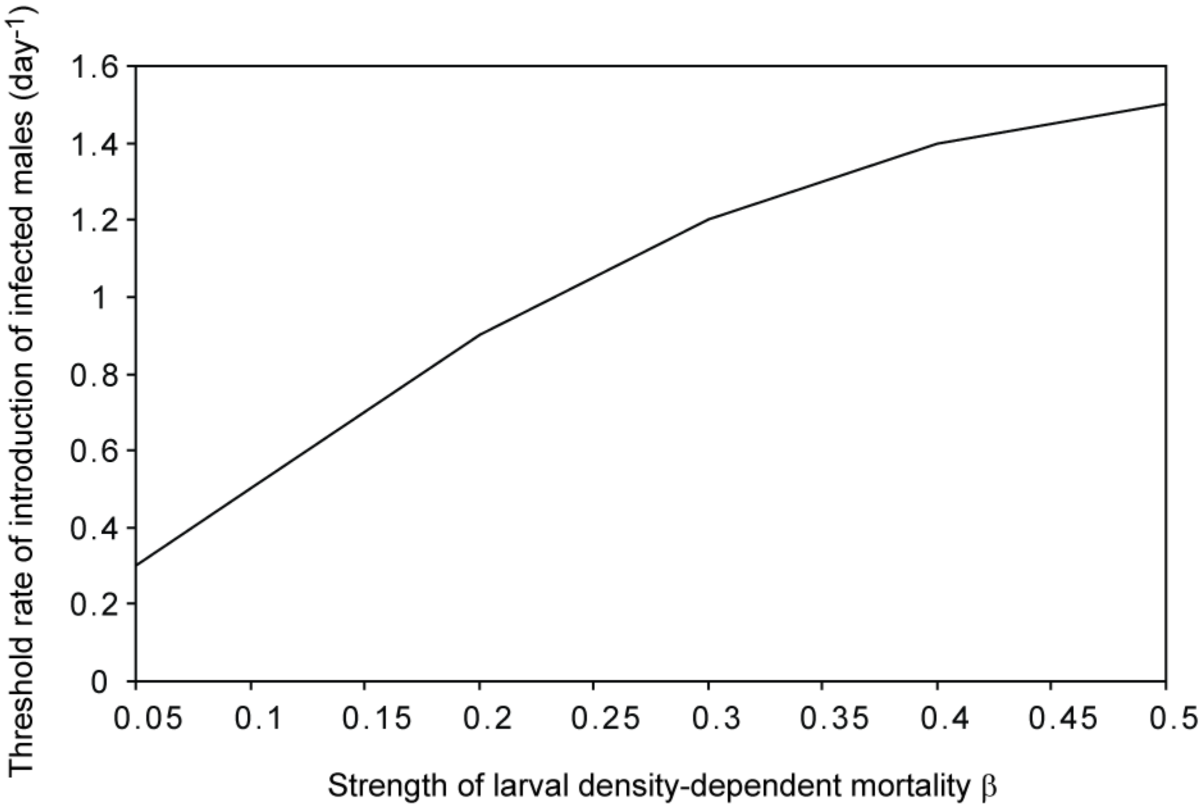

Figure S2. The minimum rate of introduction of infected males required for *Wolbachia* to spread as a function of the strength of juvenile density dependent mortality. Values of the larval carrying capacity α are chosen so that the equilibrium abundance of adults in the absence of *Wolbachia* is the same. Other parameters are as in Table 1.
