# Supplementary material for: Strategies for Introducing Wolbachia to Reduce Transmission of Mosquito-Borne Diseases
Source: PLoS Negl Trop Dis. 2011 Apr 26;5(4):e1024. doi: 10.1371/journal.pntd.0001024 (PMC3082501; doi:10.1371/journal.pntd.0001024)
Supplement: Text S2 — Modelling mosquito-borne disease transmission. (PDF) [file pntd.0001024.s002.pdf]

Strategies for introducing *Wolbachia* to reduce transmission of mosquito-borne diseases

Penelope A. Hancock, Steven P. Sinkins & H. Charles J. Godfray

Text S2: Modelling mosquito-borne disease transmission

The population dynamic model in equations (A1) and (A2) (see Text S1) can be extended to an SEI (Susceptible-Exposed-Infectious) model of disease transmission by a mosquito population. Let a certain fraction x of the human population be infectious with the disease. This model assumes that mosquitoes bite humans at a daily rate f, that disease is transmitted from human to mosquito with probability b. Once ingested by the mosquito during an infectious blood meal, the pathogen requires a period of incubation before it can be transmitted to humans. We assume this period is of fixed duration T_E. The probability of transmission of the pathogen from an infectious mosquito to a human during a bloodmeal is c. We assume that a proportion c_w of the mosquitoes that are infected with both the *Wolbachia* and the pathogen do not become infectious. This represents the reduction in disease transmission that has recently been shown to occur in mosquitoes infected with *Wolbachia* [2,4,7,25-27]. Model parameters are given in Table 1.

In modelling disease transmission we now consider only the female mosquito population as males do not transmit disease. The malaria transmission model divides the total adult female population into classes of mosquitoes that are susceptible, exposed (incubating the pathogen), and infectious. The model is of similar form to that of Hancock et al. [21]. Consider firstly the population of females uninfected with *Wolbachia.* The number of susceptible adults of age a at time t is.

$$S_{UF}(t,a) = A_{UF}(t,a)\,\theta_S(a) \qquad \text{(B1)}$$

where $\theta_S(a)$ is the probability of not contracting malaria in time a, e^{-fbxa}. To obtain the number of exposed adults we need to specify the amount of time since contracting the pathogen, n. The number of exposed adults of age a at time t that contracted the pathogen n days ago is

$$E_{UF}(t,n,a) = S_{UF}(t-n,a-n)\,fbx\theta_E(a-n,a), \qquad a \geq n, \qquad n \leq T_E \qquad \text{(B2)}$$

where $\theta_E(a-n,a)$ is the probability of surviving from age a-n to age a, which is $\theta_{A,U}(a-n)/\theta_{A,U}(a)$.

The total number of infectious adults uninfected with *Wolbachia* at time t is then

$$\tilde{I}_{UF}(t) = \tilde{A}_{UF}(t) - \tilde{S}_{UF}(t) - \tilde{E}_{UF}(t) \qquad \text{(B3)}$$

where the total number of susceptible and exposed adults, $\tilde{S}_{UF}(t)$ and $\tilde{E}_{UF}(t)$ are calculated by integrating eqn (B1) over all ages *a* and eqn (B2) over *n* and *a* using the specified limits. If we assume that the rate at which *Wolbachia* infected females are introduced into the uninfected population is negligible ($I_F(t) \to 0$) then the same equations can be used to obtain the number of adult females that are infected with *Wolbachia* and infectious with the disease at time *t*, $\tilde{I}_{WF}(t)$. We also need to account for the reduction in the number of *Wolbachia* infected mosquitoes that are infectious. This gives

$$\tilde{I}_{WF}(t) = c_W(\tilde{A}_{WF}(t) - \tilde{S}_{WF}(t) - \tilde{E}_{WF}(t)) \quad \text{(B4)}$$

We can now calculate the daily EIR, which is the number of bites by infectious mosquitoes per person per day [22]. This is

$$EIR(t) = \frac{mf}{H}(I_{UF}(t) + I_{WF}(t)) \quad \text{(B5)}$$

where *H* is the number of humans.

Equilibria

Bonsider firstly the population of females uninfected with *Wolbachia*. The equilibrium abundance of susceptible females is equal to the equilibrium total female population size multiplied by the proportion of the population that has not contracted the pathogen,

$$S^*_{UF} = A^*_{UF} \frac{\int_0^\infty \theta_{A,U}(a)da}{\int_0^\infty \theta_S(a)\theta_{A,U}(a)da} \quad \text{(B6)}$$

The equilibrium total number of adults uninfected with *Wolbachia* is $(1 - p^*_M)A^*_{WF} / p^*_M$, where the expression for the equilibrium total number of infected females, A^*_{WF}, is given by eqn (A14) of Text S1. The equilibrium abundance of exposed females is equal to the equilibrium abundance of susceptibles multiplied by the proportion that contract malaria and whose malaria infection age is less than T_E,

$$E^*_{UF} = S^*_{UF} fbx \frac{\int_0^{T_E}\int_p^\infty \theta_S(a-p)\theta_{A,U}(a)dadp}{\int_0^\infty \theta_S(a)\theta_{A,U}(a)da} \quad \text{(B7)}$$

The equilibrium abundance of infectious females is then

$$I^*_{UF} = A^*_{UF} - S^*_{UF} - E^*_{UF} \quad \text{(B8)}$$

The same procedure can be used to calculate the equilibrium number of infectious females that carry *Wolbachia*, with

$$I_{WF}^{*} = c_{w}(A_{WF}^{*} - S_{WF}^{*} - E_{WF}^{*}) \tag{B9}$$

The equilibrium EIR is then

$$EIR^{*} = \frac{ma}{H}(I_{UF}^{*} + I_{WF}^{*})\,. \tag{B10}$$
