# Supplementary material for: Strategies for Introducing Wolbachia to Reduce Transmission of Mosquito-Borne Diseases
Source: PLoS Negl Trop Dis. 2011 Apr 26;5(4):e1024. doi: 10.1371/journal.pntd.0001024 (PMC3082501; doi:10.1371/journal.pntd.0001024)
Supplement: Text S3 — Modelling adult age-dependent mortality. (PDF) [file pntd.0001024.s003.pdf]

Strategies for introducing *Wolbachia* to reduce transmission of mosquito-borne diseases

Penelope A. Hancock, Steven P. Sinkins & H. Charles J. Godfray

Text S3: Modelling adult age-dependent mortality

Similar to Hancock et al. [21], age-dependent mortality in adult mosquitoes is modelled using a Weibull function. Adults are also assumed to experience a 'background' risk of mortality that is independent of age. In mosquitoes uninfected with *Wolbachia*, adult survival to age a is given by

$$\theta_{A,U}(a) = \exp - \int_0^a \mu_{A,U}(\tau) d\tau; \qquad \mu_{A,U}(a) = c_U + \gamma_U r_U (r_U a_U)^{\gamma_U - 1} \qquad \text{(C1)}$$

where $\mu_{A,U}(a)$ is the daily rate of adult mortality, c_U is a constant term representing age-independent mortality and γ_U and r_U are the shape and rate parameters of the Weibull function representing age-dependent mortality. Mortality in adult mosquitoes infected with *Wolbachia* is described by the same function with different parameters, replacing the subscript U with subscript W.

Parameterising adult age-dependent mortality

We use laboratory survival data for *Anopheles stephensi* [23] to estimate the parameters of the Weibull function describing age-dependent adult mortality in mosquitoes uninfected with *Wolbachia*, γ_U and r_U (eqn C1). To incorporate the additional mortality experienced by mosquitoes in nature, we set the constant term c_U in eqn (C1) to a value typically recorded for field populations of *Anopheles* (Table 1).

We use data from laboratory colonies of *Aedes aegypti* infected with the life-shortening *Wolbachia* strain *w*MelPop to estimate the effect of *Wolbachia* infection on adult mortality [6]. For a given value of the Weibull shape parameter ($\gamma_U = \gamma_W$) we found that the effect of *w*MelPop infection on adult survival could be well-approximated by doubling the Weibull rate parameter (r_W=2 r_U) (Table 1). Both infected and uninfected adults are assumed to experience the same risk of 'background' age-independent mortality ($c_U = c_W$).
