# Supplementary material for: Strategies for Introducing Wolbachia to Reduce Transmission of Mosquito-Borne Diseases
Source: PLoS Negl Trop Dis. 2011 Apr 26;5(4):e1024. doi: 10.1371/journal.pntd.0001024 (PMC3082501; doi:10.1371/journal.pntd.0001024)
Supplement: Text S4 — The effect of juvenile density-dependent mortality on the required number of released females. (PDF) [file pntd.0001024.s004.pdf]

Strategies for introducing *Wolbachia* to reduce transmission of mosquito-borne diseases

Penelope A. Hancock, Steven P. Sinkins & H. Charles J. Godfray

Text S4: The effect of juvenile density-dependent mortality on the required number of released females.

From the results in Figure 2A, showing *Wolbachia* introduction into a seasonally fluctuating mosquito population, we concluded that male-biased releases require between ½-⅓ the number of females to be introduced compared to equal sex-ratio releases, across a range of different release times. Here we explore how the form of juvenile density-dependent mortality affects this conclusion. For each release sex-ratio the required releases sizes were compared for two populations, one in which density-dependence is relatively strong (β=0.3) and the other in which it is weaker (β=0.1). For the population with strong density-dependence, α was set to 0.019 during the six month high abundance period and 0.038 for the rest of the year. For the population with weaker density-dependence, α was set to 0.13 during the six month high abundance period and 0.19 for the rest of the year. These values of α were specified so that the total annual adult recruitment in the absence of *Wolbachia* was the same for both populations. However the pattern of seasonal variation in mosquito abundance differs between the different density-dependence forms (Figure S3).

Assuming a release strategy of 30 daily releases, Figure S3 compares the minimum required release size, and the corresponding number of introduced females, for equal sex-ratio and 95% male-biased releases for weak (Figure S3A) and strong (Figure S3B) density-dependence. For both forms of density-dependence ½-⅓ the number of females was required for male-biased compared to equal sex-ratio releases, across all release times.

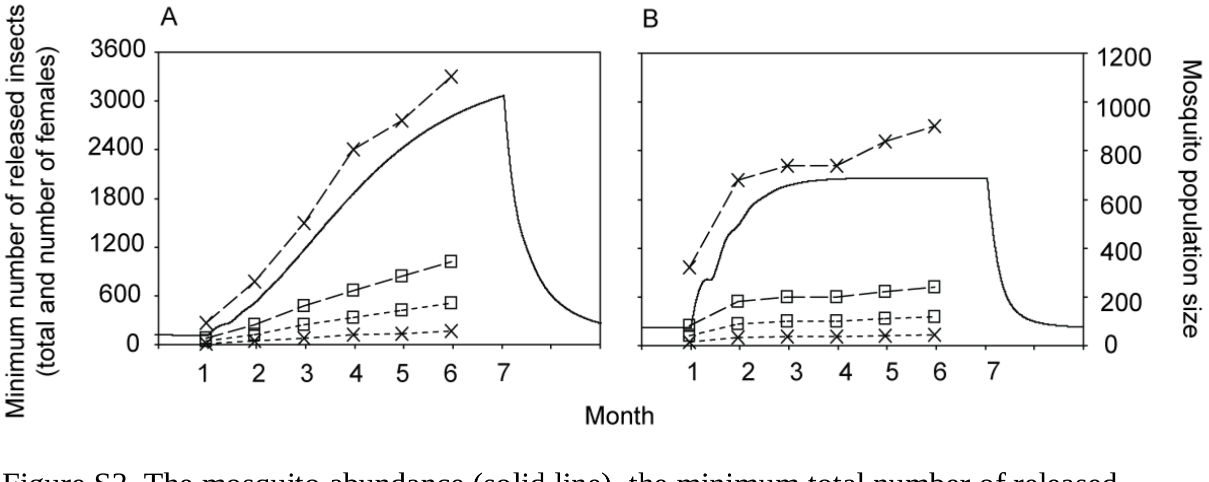

Figure S3. The mosquito abundance (solid line), the minimum total number of released mosquitoes required for *Wolbachia* spread (dashed lines), and the number of released females (dotted lines) for different release times. Open squares show equal sex ratio releases and

crosses show 95% male releases. Panels A and B show weak (β=0.1) and strong (β=0.3) density-dependence respectively. Values of the larval carrying capacity α are specified as described in the text, and other parameters are as in Table 1.
