# Supplementary material for: Strategies for Introducing Wolbachia to Reduce Transmission of Mosquito-Borne Diseases
Source: PLoS Negl Trop Dis. 2011 Apr 26;5(4):e1024. doi: 10.1371/journal.pntd.0001024 (PMC3082501; doi:10.1371/journal.pntd.0001024)
Supplement: Text S5 — The effect of varying the number of releases on the required release size. (PDF) [file pntd.0001024.s005.pdf]

Strategies for introducing *Wolbachia* to reduce transmission of mosquito-borne diseases

Penelope A. Hancock, Steven P. Sinkins & H. Charles J. Godfray

Text S5: The effect of varying the number of releases on the required release size

For the case where mosquitoes are released in a 95% male sex ratio we explored whether the total number of released mosquitoes required for *Wolbachia* spread was affected by the number of releases made. Release strategies of making a single release and 15 and 30 daily releases were compared. Mosquito abundance dynamics were assumed to follow seasonal pattern A. Figure S4 shows that for releases made towards the middle of the high abundance season after the period of rapid population growth, single releases require more mosquitoes to be released in total than multiple releases (15 or 30 batches). However there is no advantage to making multiple releases when the population is growing rapidly early in the season. Seasonal variation in mosquito abundance is a much stronger determinant of the required release size than the number of releases made.

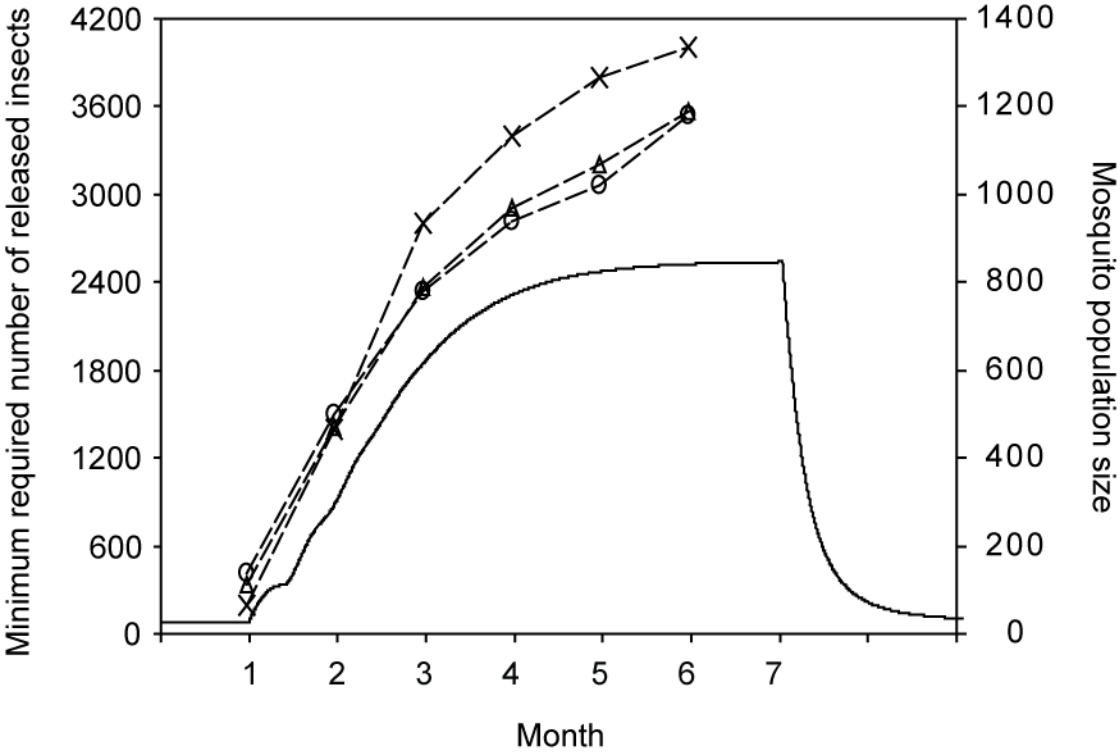

Figure S4. The mosquito population size (solid line) and the minimum total number of mosquitoes required for *Wolbachia* to spread (dashed lines). Open circles, open triangles and crosses show release strategies of 30 daily releases, 15 daily releases and a single release respectively. Mosquito population size follows seasonal pattern A and releases have a 95% male sex ratio. Other parameters are as in Table 1.
