# Supplementary material for: Strategies for Introducing Wolbachia to Reduce Transmission of Mosquito-Borne Diseases
Source: PLoS Negl Trop Dis. 2011 Apr 26;5(4):e1024. doi: 10.1371/journal.pntd.0001024 (PMC3082501; doi:10.1371/journal.pntd.0001024)
Supplement: Text S6 — The effect of male-biased Wolbachia release on female population size and EIR for different forms of juvenile density-dependence. (PDF) [file pntd.0001024.s006.pdf]

# Strategies for introducing *Wolbachia* to reduce transmission of mosquito-borne diseases

Penelope A. Hancock, Steven P. Sinkins & H. Charles J. Godfray

## Text S6: The effect of male-biased *Wolbachia* release on female population size and EIR for different forms of juvenile density-dependence

From the results in Figures 3 and 4 we concluded that male-biased release of *Wolbachia* infected insects causes an overall reduction in the female population size and the EIR in the year of release, although a small, brief increase in both may occur at the time of the releases. In the longer term once *Wolbachia* has spread to a high frequency, there is a strong further decline in the EIR. Here we explore how these conclusions are influenced by the assumed form of juvenile density-dependent mortality. Again we compare the effects of *Wolbachia* release for two seasonally fluctuating mosquito populations, one in which density dependence is strong and the other in which it is relatively weak. The parameterisation for each case is the same as that described in Text S4. We examine the variation in the EIR and the female population size over a 3 year period, where 30 daily 95% male releases of the minimum total number necessary for *Wolbachia* spread are made in the second year in the second month of the high abundance season.

Figures S5A & B show the results for weak and strong density-dependence respectively. When density-dependence is strong, *Wolbachia* release has only a minor effect on the female population size, both at the time of releases and in the longer term once *Wolbachia* has spread (Figure S5B). The reduction in female abundance is much greater when density-dependence is weaker. This is because when density-dependence is strong the rate of juvenile mortality is greatly reduced as a result of the drop in the numbers of larvae caused by offspring mortality due to incompatible matings and the fitness costs of *Wolbachia* in infected females. Recruitment to the adult population is therefore relatively unaffected. However, the spread of *Wolbachia* to a high frequency causes a very similar reduction in the EIR for both forms of density-dependence (Figure S5). This is because the EIR is much more sensitive to a reduction in vector lifespan than to suppression of the rate of adult recruitment.

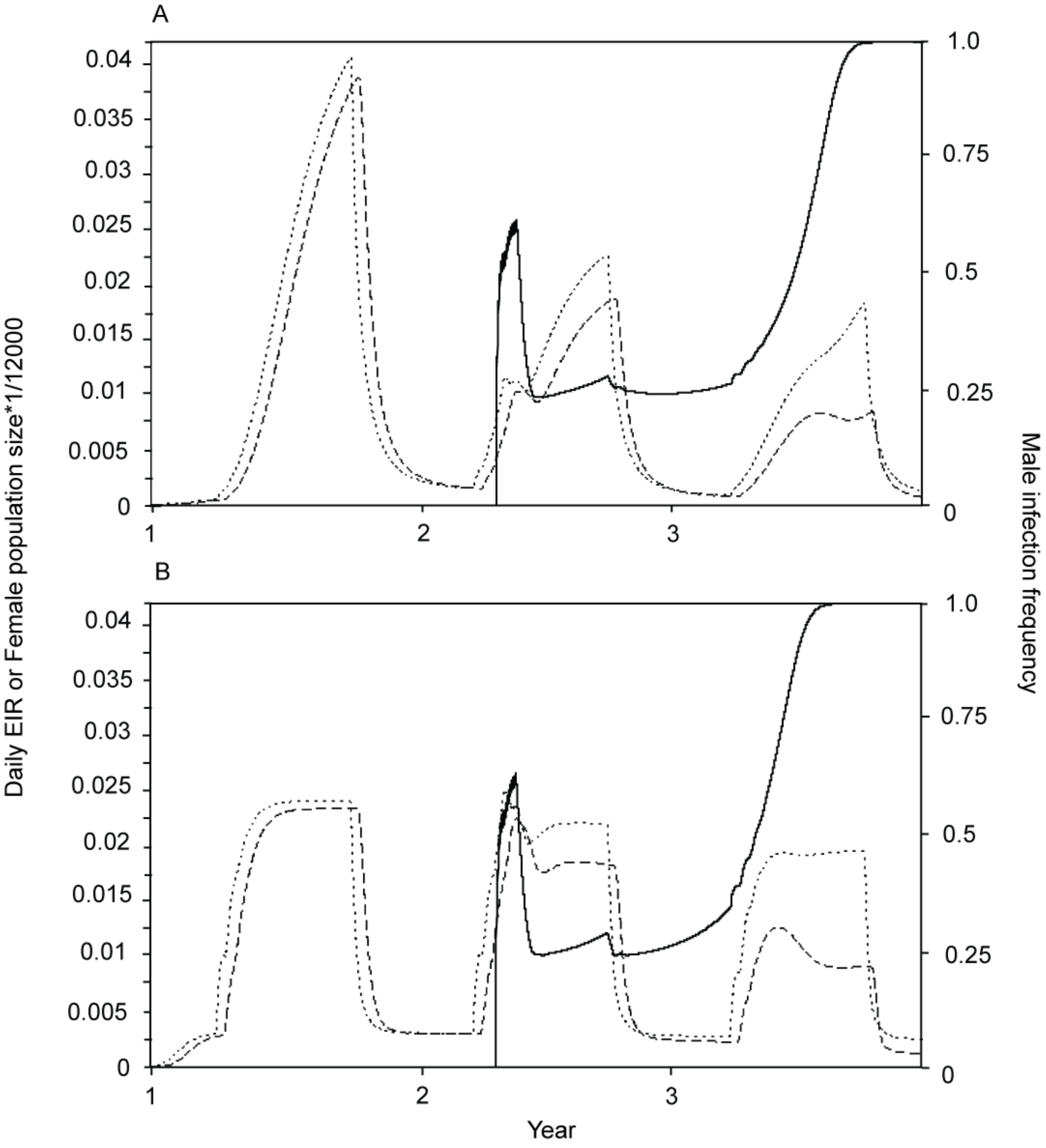


Figure S5. The daily EIR (dashed line), the female population size (dotted line) and the male infection frequency (solid line) as a function of time following 30 daily 95% male releases of the minimum number of *Wolbachia*-infected mosquitoes required for spread. Releases begin in the second year in the second month of the high-abundance season. Seasonal dynamics follow pattern A. Panels A and B show weak ($\beta$=0.1) and strong ($\beta$=0.3) density-dependence respectively. Values of the larval carrying capacity $\alpha$ are specified as described in the text, and other parameters are as in Table 1.
