# Supplementary material for: Strategies for Introducing Wolbachia to Reduce Transmission of Mosquito-Borne Diseases
Source: PLoS Negl Trop Dis. 2011 Apr 26;5(4):e1024. doi: 10.1371/journal.pntd.0001024 (PMC3082501; doi:10.1371/journal.pntd.0001024)
Supplement: Text S7 — Effects of equal sex ratio releases on the EIR and female population size. (PDF) [file pntd.0001024.s007.pdf]

Strategies for introducing *Wolbachia* to reduce transmission of mosquito-borne diseases

Penelope A. Hancock, Steven P. Sinkins & H. Charles J. Godfray

Text S7: Effects of equal sex ratio releases on the EIR and female population size

This study has shown the effect of 95% male-biased releases of *Wolbachia* infected insects on the EIR and the abundance of female mosquitoes, assuming that the mosquito population size fluctuates seasonally and that releases are made near the beginning of the high abundance season (Figures 4 & 5). We now demonstrate the effect of equal sex ratio releases, again considering the same two release strategies: (i) introduction of the minimum number of mosquitoes required for *Wolbachia* spread in 30 daily releases; (ii) introduction of three times the minimum required number for spread in 90 daily releases. Both release strategies cause only minor increases in the female population size at the time of release compared to the natural level, which is similar to the result obtained for male-biased releases (Figure S6A & B). However equal sex ratio releases cause much less transient suppression of the female numbers and the EIR throughout the process of *Wolbachia* spread than male-biased releases. This is because equal sex ratio releases add more females to the population than male-biased releases, and they cause much less artificial elevation of the male infection frequency.

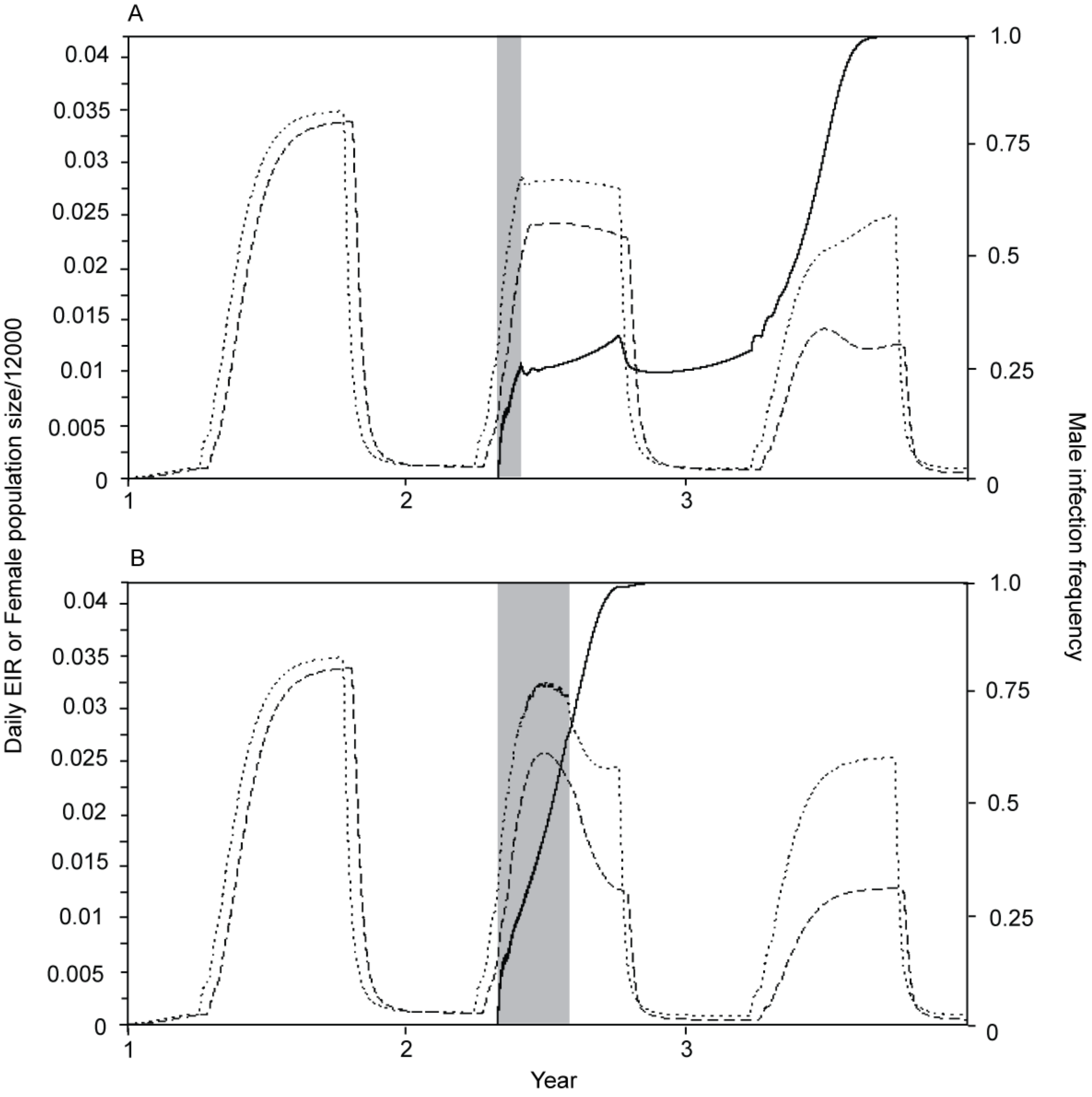

Figure S6. The daily EIR (dashed line) and the female population size (dotted line) following equal sex ratio releases of *Wolbachia*-infected mosquitoes. A. The minimum total number required for spread are released in 30 daily releases; B. Three times the minimum total number required for spread are released in 90 daily releases. Releases begin in the second year in the second month of the high-abundance season. Seasonal dynamics follow pattern A. The solid line shows the male infection frequency and the shaded area shows the period during which releases occur. Other parameters are as in Table 1.
