# Supplementary material for: Strategies for Introducing Wolbachia to Reduce Transmission of Mosquito-Borne Diseases
Source: PLoS Negl Trop Dis. 2011 Apr 26;5(4):e1024. doi: 10.1371/journal.pntd.0001024 (PMC3082501; doi:10.1371/journal.pntd.0001024)
Supplement: Text S8 — Wolbachia introduction following pre-release population suppression. (PDF) [file pntd.0001024.s008.pdf]

Strategies for introducing *Wolbachia* to reduce transmission of mosquito-borne diseases

Penelope A. Hancock, Steven P. Sinkins & H. Charles J. Godfray

Text S8: *Wolbachia* introduction following pre-release population suppression

The model can be used to explore strategies of artificially suppressing the natural mosquito population prior to *Wolbachia* release. Here it is assumed that the pre-release suppression increases the rate of adult mortality, which is modelled by incrementing the value of c_U, the constant term representing the rate of age-independent mortality (see eqn (C1) of Text S3). The pre-release control is assumed to last for 1 month immediately prior to the first release of *Wolbachia*-infected mosquitoes, at which point they are stopped. At this time 30 daily releases of equal numbers of male and female mosquitoes infected with *Wolbachia* are initiated. Seasonal variation in mosquito abundance is ignored for simplicity.

Figure S8 shows how the pre-release suppression decreases the minimum number of released females required for *Wolbachia* to spread. If the pre-release suppression achieves very large reductions in the adult population size at the time of the first release, the required number of released females can be lessened by around 60%. If pre-release suppression is less effective considerably more females need to be introduced.

The results indicate that pre-release population suppression may not greatly reduce the number of introduced infected insects required for *Wolbachia* to spread unless it has a major impact on adult abundance. This is because the population can recover quickly after the suppression measures cease because the population size is below the carrying capacity and juveniles experience relatively low competition. However, pre-release suppression will clearly help to avoid possible increases in the rates of human biting and disease transmission above their natural levels following *Wolbachia* release.

The results in the main text showed that when the releases have a 95% male sex-ratio the required number of introduced females is between ⅓-½ the number needed in the equal sex-ratio strategy. This suggests that a strategy of male-biased releases is likely to require fewer introduced females than a strategy of pre-release population suppression followed by equal sex-ratio releases. Further, male-biased releases can be continued for an extended period of time until *Wolbachia* spreads without increasing the abundance of females.

However the efficacy of pre-release suppression in reducing the required release size will depend on the number and frequency of the releases. For example if the insects are released in a small number of large releases, *Wolbachia* spread may occur before the population has had time to fully recover from the pre-release suppression. However making large releases can be disadvantageous because they have a strong effect on the level of density-dependent competition experienced by the population (see Hancock et al. [14]).

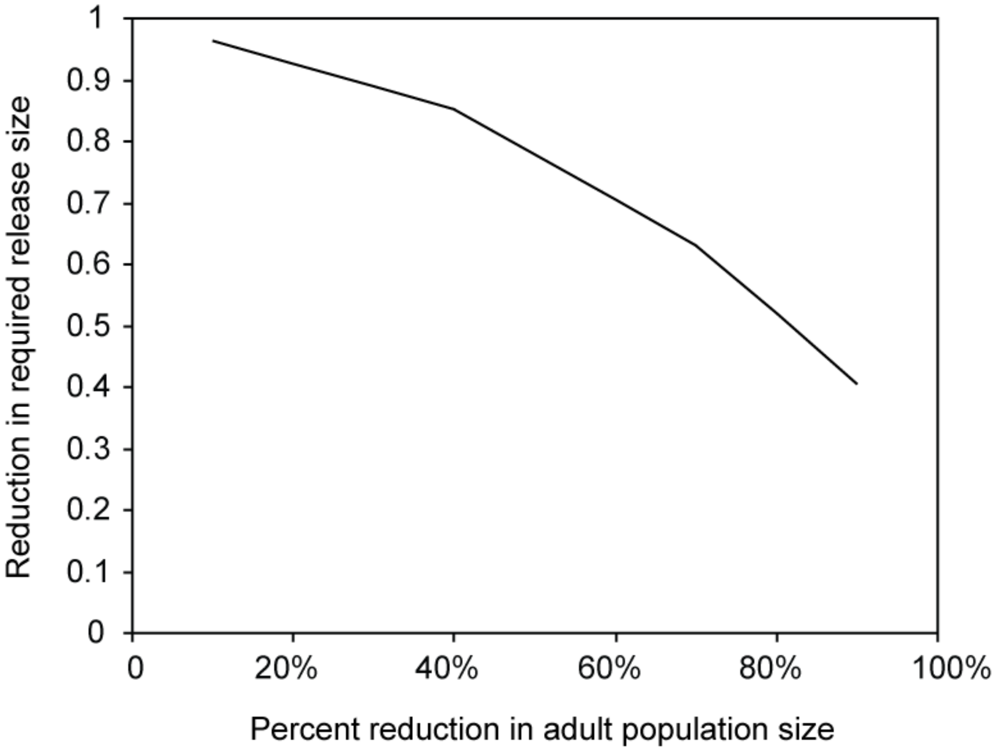

Figure S8. The proportional reduction in the minimum number of released females required for *Wolbachia* to spread for different percentage reductions in the adult abundance achieved by pre-release suppression at the time of the first release. Pre-release suppression is achieved by killing adults, increasing the rate of age-independent adult mortality c_U (see text). After the pre-release suppression, 30 daily releases of equal numbers of male and female mosquitoes infected with *Wolbachia* are initiated. Other parameters are as in Table 1.
